# Standardizing the Clinical Approach to Cancer Therapy‐Related Cardiac Dysfunction: Applying Cardio‐Oncology Guidelines as a Practical Tool for Hematology and Oncology Providers

**DOI:** 10.1002/cam4.71682

**Published:** 2026-03-27

**Authors:** Abdelrahman Ali, Suparna C. Clasen, Anne Blaes, Stephen Casselli, Anita Deswal, Susan Halli Demeter, Gregory Durm, Anecita Fadol, Alessandra Ferrajoli, Michael G. Fradley, Joerg Herrmann, Borja Ibanez, Sue Koob, Bogda Koczwara, Kasey J. Leger, Jennifer E. Liu, Teresa López‐Fernández, Alexander R. Lyon, N. G. Choon Ta, John Teerlink, Eric H. Yang, Susan Dent, Daniel Lenihan

**Affiliations:** ^1^ Division of Cardiology University of Texas Medical Branch Galveston Texas USA; ^2^ Division of Cardiology, Department of Medicine Indiana University School of Medicine Indianapolis Indiana USA; ^3^ Division of Hematology, Oncology and Transplantation University of Minnesota Minneapolis Minnesota USA; ^4^ International Cardio‐Oncology Society Tampa Florida USA; ^5^ Department of Cardiology University of Texas at MD Anderson Cancer Center Houston Texas USA; ^6^ Department of Cardiovascular Diseases Mayo Clinic Scottsdale Arizona USA; ^7^ Indiana University Simon Comprehensive Cancer Center Indianapolis Indiana USA; ^8^ Department of Leukemia, Division of Cancer Medicine University of Texas at MD Anderson Cancer Center Houston Texas USA; ^9^ Division of Cardiology Hospital of the University of Pennsylvania Philadelphia Pennsylvania USA; ^10^ Department of Cardiovascular Medicine Mayo Clinic Rochester Minnesota USA; ^11^ Centro Nacional de Investigaciones Cardiovasculares (CNIC) & IIS Fundación Jiménez Díaz, UAM & CIBERCV Madrid Spain; ^12^ Preventive Cardiovascular Nurses Association Madison Wisconsin USA; ^13^ Australian Research Centre for Cancer Survivorship, University of New South Wales, Sydney Australia, and Flinders Health and Medical Research Institute, Flinders University Bedford Park Australia; ^14^ Seattle Children's Hospital, University of Washington Seattle Washington USA; ^15^ Cardiology Service, Memorial Sloan Kettering Cancer Center New York New York USA; ^16^ Cardiology Department La Paz University Hospital, IdiPAZ Research Institute Madrid Spain; ^17^ Cardio‐Oncology Centre of Excellence Royal Brompton Hospital London UK; ^18^ Department of Cardiology National Heart Centre Singapore Singapore Singapore; ^19^ Section of Cardiology, San Francisco Veterans Affairs Medical Center San Francisco California USA; ^20^ Division of Cardiology, Department of Medicine University of California at Los Angeles Los Angeles California USA; ^21^ Department of Medicine Wilmot Cancer Institute, University of Rochester Rochester New York USA; ^22^ Saint Francis Healthcare Cape Girardeau Missouri USA

**Keywords:** cancer therapy–related cardiac dysfunction, cardiotoxicity, risk stratification, surveillance

## Abstract

**Introduction and Methods:**

Cancer therapy–related cardiac dysfunction (CTRCD) is a well established and potentially life‐threatening complication of contemporary oncologic treatment. Although comprehensive cardio‐oncology guidelines have been developed, their integration into routine hematology and oncology practice remains inconsistent. This consensus statement, developed by a multidisciplinary panel of cardio‐oncology experts, aims to provide practical, case‐based guidance to help oncology providers recognize, assess, and manage CTRCD across a spectrum of malignancies and cardiovascular presentations.

**Clinical Scenarios and Discussion:**

We present representative clinical scenarios that illustrate real‐world challenges in cardio‐oncology and apply evidence‐based recommendations from current guidelines, including those from the European Society of Cardiology (ESC) and the International Cardio‐Oncology Society (ICOS)—to support informed decision‐making. Key areas of focus include baseline cardiovascular risk stratification prior to initiating potentially cardiotoxic therapies, with an emphasis on biomarker and imaging surveillance strategies tailored to individual risk profiles. Also, this document outlines the application of guideline‐directed medical therapy (GDMT) for cancer patients with heart failure.

**Conclusion:**

By offering a structured, user‐friendly framework, this document seeks to bridge the implementation gap between oncology and cardiology disciplines. Our goal is to equip oncology providers with accessible tools that facilitate early recognition, consistent surveillance, and timely referral, thereby preserving cancer treatment intensity while minimizing cardiovascular morbidity.

**Abbreviations**
ACE‐I: angiotensin‐converting enzyme (ACE) inhibitor
ARB: angiotensin II receptor blocker
ARNI: angiotensin receptor neprilysin inhibitor
BB: beta‐blocker (specifically those preferred in systolic HF: carvedilol, metoprolol XL, bisoprolol)
CAD: coronary artery disease
CMR: cardiac magnetic resonance
CO: Cardio‐Oncology
CTRCD: cancer therapy‐related cardiac dysfunction
CV: Cardiovascular
DM: diabetes mellites
GDMT: guideline directed medical therapy
GLS: global longitudinal strain
HF: heart failure
HL: hyperlipidemia
HTN: hypertension
ICI: immune checkpoint inhibitors
IC‐OS: international cardio‐oncology society
LVEF: left ventricular ejection fraction
NT‐proBNP: N‐terminal pro‐B type natriuretic peptide
SGLT2i: sodium‐glucose transport protein 2 inhibitors

## 1 Introduction

How Can the Cardio‐Oncology Guidelines Regarding Cancer Therapy‐Related Cardiac Dysfunction (CTRCD) be Utilized Effectively in Practice?

Recent publications have established standard definitions of cardiac dysfunction during cancer therapy by incorporating a variety of professional society‐based recommendations. Efforts are being made to consistently report cardiovascular toxicity (CV) encountered by patients during cancer therapy, but these efforts as well as the optimal management of the identified toxicities are not well established. The range of CV toxicities has become increasingly diverse and complicated due to the advent of numerous novel targeted treatments and immunotherapies. This widening horizon of potential CV adverse events was the impetus for developing common definitions of CV toxicity, and subsequently, the first comprehensive set of Cardio‐Oncology (C—O) guidelines predominantly for clinicians and researchers [1, 2]. This extensive guideline aimed to be an authoritative review and comprehensive compendium in this expanding field with understandable and concise recommendations for monitoring and treating toxicities based on data, when available, or in its absence based on expert consensus. Cardiac dysfunction, with or without symptomatic heart failure (HF), was the original form of “cardiotoxicity” observed, historically with anthracycline use, and is the most common form of CV toxicity overall that requires monitoring. The current position statement will discuss how monitoring for Cancer Therapy‐Related Cardiac Dysfunction (CTRCD) and optimal cardioprotective strategies for identified CTRCD can be implemented for practice improvement with early identification and timely intervention. These monitoring and treatment recommendations aim to reduce interruptions or delays in cancer therapy due to CTRCD and to detect cardiac dysfunction at its earliest stage to allow effective cardioprotective‐based therapy for longer term cardiovascular health. The unifying mission is to mitigate CV disease and CTRCD as a barrier to life‐saving cancer therapy.

Although the definition of CTRCD has varied across guidelines and consensus statements [1, 3, 4], the need for standardized criteria led to the 2022 consensus statement by the International Cardio‐Oncology Society (IC‐OS) which provides clear recommendations on defining cardiovascular toxicities contributing to CTRCD [2]. However, the impact of these recommendations on clinical practice is unclear, particularly among hematologists and oncologists, where awareness of these initiatives may not be widespread. The cancer‐treating team is the principal provider that prescribes potentially cardiotoxic therapies and is typically the first to encounter significant CV adverse events. Therefore, any guideline‐type recommendation needs to be practically targeted toward clinicians managing cancer patients.

This document aims to bridge this gap between guideline recommendation and practice by employing these standardized definitions of cardiovascular toxicity, in particular CTRCD, to five patient scenarios and focus on challenges and practical guidance for patient management.

## 2 Methodology

This consensus statement was developed by a multidisciplinary writing group of cardiology and hematology/oncology experts convened by the IC‐OS Executive Board for a Scientific Summit Committee with the following organizations providing representation: Advanced Cardiac Therapies Improving Outcomes Network (ACTION), American College of Cardiology (ACC), American Heart Association (AHA), American Society of Hematology (ASH), American Society for Transplantation and Cellular Therapy (ASTCT), European Society of Cardiology (ESC), Heart Failure Society of America (HFSA), Multinational Association of Supportive Care in Cancer (MASCC), National Comprehensive Cancer Network (NCCN), and the Preventive Cardiovascular Nurses Association (PCNA). The writing group conducted bimonthly teleconferences from January 2024 to May 2025, during which subgroups focused on specific topics and conducted extensive literature reviews.

**FIGURE 1:**
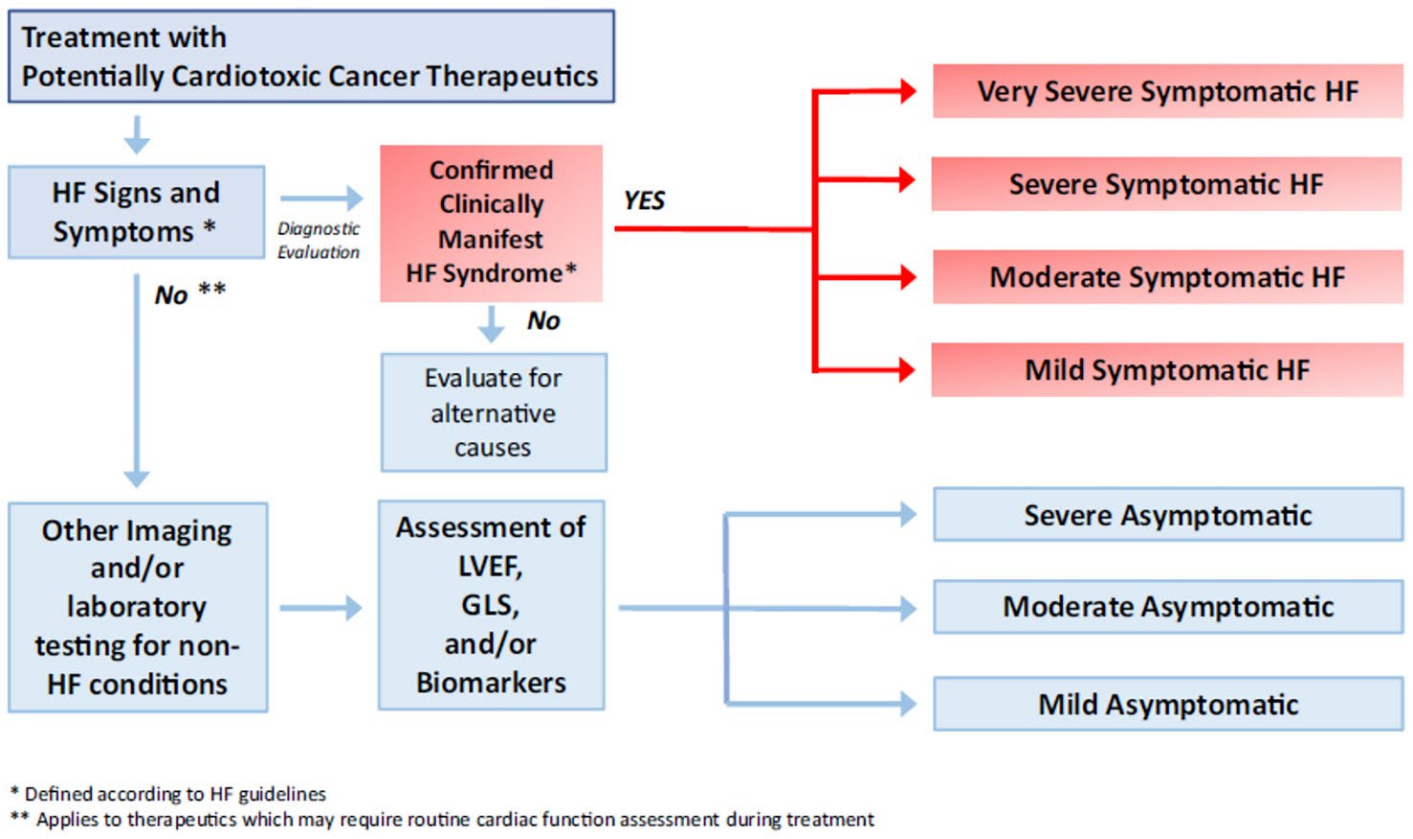
Diagnostic algorithm for cancer therapy‐related cardiac dysfunction.

CTRCD was defined according to the previously published diagnostic algorithm (Figure 1 and Table S2) [2]. Additionally, Table S1 outlines proposed screening criteria for heart failure (HF), providing a structured approach to identifying at‐risk patients in the clinical setting [5]. In addition, after each case scenario a multiple choice question was used to focus the clinician on the important decisions encountered in practice.

## 3 Clinical Scenarios

### 3.1 Breast Cancer

A 43‐year‐old pre‐menopausal female who is an active smoker presents to the C‐O clinic for evaluation of an asymptomatic decline in left ventricular ejection fraction (LVEF), identified by a follow‐up transthoracic echocardiogram (Echo). She was recently diagnosed with T1c (1.1 cm) N0M0, estrogen‐positive, progesterone‐negative, and HER2‐positive breast cancer and underwent left breast conservative surgery and sentinel lymph node biopsy. She completed adjuvant chemotherapy with 12 weeks of paclitaxel administered concurrently with trastuzumab and has since completed 6 of a planned 14 cycles of trastuzumab (given every 3 weeks).

Her baseline LVEF by echo was 58%, and during the routine recommended “every 3 months monitoring,” her LVEF declined to 43% at 6 months while global longitudinal strain (GLS) was not reported. The patient did not have any symptoms or signs of HF (Table 1). She has lost 5–10 pounds since the initiation of chemotherapy but is able to perform daily activities without limitations. She does not follow a regular exercise regimen and continues to smoke half a pack of cigarettes daily. There is no family history of cardiomyopathy or premature (CAD in women before 65 or in men before 55 years of age) coronary artery disease (CAD*).

**TABLE 1:** Symptoms and signs of heart failure.

Symptoms	Signs
Typical	More specific
Orthopnea	Elevated jugular venous pressure
Paroxysmal nocturnal dyspnea	Third heart sound (S3 gallop rhythm)
Reduced exercise tolerance	Systolic murmur
Fatigue	
Lower extremity edema	
Less typical	Less specific
Nocturnal cough	Weight gain (> 2 kg/week)
Wheezing	Peripheral edema (ankle, sacral, scortal)
Bloated feeling	Pulmonary crepitations (crackles)
Loss of appetite	Pleural effusion
	Tachycardia
	Tachypnea
	Hepatomegaly
	Ascites
	Cold extremities

On examination, her blood pressure is 140/85 mmHg, heart rate is 95 bpm, without clinical signs of volume overload (jugular venous distension (JVD)), crackles on lung auscultation, S3 gallop, abdominal distention, or lower extremity edema.

At this point in her clinical care, which of the following principles is best to follow for her ongoing cancer treatment?

- Trastuzumab should be discontinued because of cardiotoxicity, given an absolute decline in LVEF > 10% from baseline to an LVEF < 50%, and the fact she has completed half of her planned therapy.
- A more complete CV assessment is needed to confirm cardiotoxicity.
- Cardioprotective therapy with RAAS inhibitors or carvedilol has not been established to help in this situation.
- Continuing trastuzumab without interruption while rapidly optimizing HF cardioprotective therapy and increasing surveillance frequency to ensure LVEF remains acceptable is a reasonable approach.

Answer: The correct answer is D.

This patient has evidence of moderate asymptomatic CTRCD. The patient has a LVEF decline from 58% to 43% during HER2‐targeted therapy and meets the criteria for moderate CTRCD. According to the CTCAE criteria, this condition would be classified as CTCAE Grade 2 left ventricular dysfunction (Table S2). Discontinuation of adjuvant HER2‐targeted therapy is not recommended in asymptomatic patients, as the benefits of treatment continuation outweigh the risks—provided cardioprotective medications are initiated and close cardiac monitoring is implemented during trastuzumab therapy. In some cases, (neo) adjuvant treatment can decrease the risk of breast cancer recurrence by nearly 50% when given for 1 year for patients with HER2 positive early‐stage breast cancer [6, 7] In a small phase I trial, the SCHOLAR (Safety of Continuing Trastuzumab Despite Mild Cardiotoxicity) team evaluated the safety of continuing trastuzumab treatment in the presence of mild cardiotoxicity (LVEF ≥ 40%), while treating patients with angiotensin‐converting enzyme inhibitors (ACE‐I) and beta‐blockers (BB). Their findings suggest that it could be feasible to maintain trastuzumab therapy under these conditions. The vast majority of participants—specifically 90%—were able to complete their planned cancer therapy without experiencing significant cardiac events [7]. The SAFE‐HEaRt trial showed that it is safe to continue HER2‐targeted therapies in patients with a mildly reduced LVEF of 40%–49% while having no symptoms of HF, and initiating optimal cardioprotection with ACE‐I and carvedilol [8] Additionally, long‐term follow‐up data demonstrated that late development of cardiac dysfunction and HF is uncommon [9].

Performing a thorough history and physical examination to assess CV risk factors in patients receiving cardiotoxic therapies, such as trastuzumab, is essential [10]. Key baseline assessments should include a lipid panel, Hemoglobin A1C, and an echo including LVEF and Global Longitudinal Strain (GLS) (Class 1C). For high and very high‐risk patients, monitoring cardiac biomarkers can be useful, specifically NT‐proBNP and cardiac troponins (Class IB) [1]. Counseling for smoking cessation is necessary to reduce modifiable CV risks. Obtaining baseline NT‐proBNP value is recommended, as this biomarker can guide future therapy if the patient becomes symptomatic and holds prognostic significance (Table 2) [14].

**TABLE 2:** Recommended baseline screening evaluation for patients undergoing HER2‐targeted therapy.

	NCCN	ASCO [11]	ESMO [12, 13]	ESC/IC‐OS [1]
BNP/NT‐proBNP	NR	NR	NR	1c/IIb^a^
12‐lead ECG	NR	NR	NR	Class I, LOE C
Echo	NR	NR	NR	Class I, LOE B

Abbreviation: NR, not reported.

^a^ Class 1C in high/very high‐risk and IIb in low‐risk patients prior to anti‐HER2 therapy.

The principle of optimal cardio‐protection includes the treatment of hypertension (HTN) using therapies that are effective in preventing LV dysfunction, such as RAAS inhibitors. Elevated systolic and diastolic blood pressures are significant contributors to symptomatic HF development. It is a Class IA recommendation in the 2022 AHA/ACC/HFSA HF Guidelines to control HTN in accordance with guideline‐directed medical therapy (GDMT) [15] Additionally, modifiable cardiovascular risk factors—such as smoking, physical inactivity, along with a history of CAD and prior anthracycline exposure, are important predictors of CTRCD development in patients with cancer. Recent data confirm that regular exercise is beneficial for delaying tumor progression, enhancing overall cardiovascular fitness and improving tolerance to the cardiotoxic effects of cancer therapy [16, 17, 18, 19].

### 3.2 Lung Cancer

A 75‐year‐old male with a history of HTN, insulin‐dependent type II diabetes mellitus (DM), hyperlipidemia (HL), coronary artery disease (CAD), and a past history of 30‐pack‐year cigarette smoking presents for evaluation of progressive shortness of breath and a 15‐pound weight gain over the past 4 weeks. The patient has a known diagnosis of metastatic adenocarcinoma of the lung involving spread to the liver and adrenal glands, identified with an epidermal growth factor receptor (EGFR) exon 19 deletion mutation, receiving EGFR targeted kinase inhibitor, osimertinib 80 mg orally, for the past 6 months. No recent (within the past year) cardiac evaluation was performed prior to initiating cancer therapy.

In the 4 weeks prior to presentation, the patient has experienced worsening shortness of breath on exertion, significantly limiting his ability to perform daily activities. Additionally, he reports two‐pillow orthopnea and bilateral lower extremity edema extending to the knees. He denies chest pain, syncope, or palpitations. His blood pressure is 140/90 mmHg, and the oxygen saturation is 94%. Current medications include aspirin 81 mg, amlodipine 5 mg, metformin 500 mg twice daily, insulin, and pravastatin 20 mg daily. A recent follow‐up FDG‐PET scan showed improvement in the FDG avid mass within the left lower lobe and improving FDG avid left cervical, axillary, hilar, and mediastinal lymph nodes. An electrocardiogram (ECG) demonstrated normal sinus rhythm with a heart rate of 85 bpm, a QRS duration of 100 ms, and a QTc of 450 ms. An echo ordered by his oncologist revealed an LVEF of 38%, grade II diastolic dysfunction with elevated filling pressures, a dilated inferior vena cava, borderline elevated pulmonary artery systolic pressure estimate of 38 mmHg, and a dilated left atrium.

Do the echocardiographic findings influence ongoing cancer therapy? What are the next steps?

- Hold osimertinib, check NT‐proBNP level, and repeat echo in 6 weeks.
- Continue osimertinib at reduced dose (40 mg).
- Continue osimertinib at current dose, repeat echo in 4–6 weeks.
- Hold osimertinib, check cardiac biomarkers, consider ischemic evaluation, optimize medications for the treatment of HF, and reassess LV function in 4–6 weeks.

The correct answer is D. This patient presents with acute decompensated HF with reduced LVEF. Given the patient's presentation, he would be presumed to have osimertinib‐associated CTRCD and classified as severe symptomatic (although baseline LVEF prior to initiating therapy is not known). According to the CTCAE Version 5 criteria, this corresponds to grade 3 HF and grade 3 reduced LVEF. The first step in management would be to hold osimertinib due to the severe LV dysfunction/HF. Evidence on the reversibility of osimertinib‐associated cardiomyopathy and the potential for successful rechallenge, even at reduced doses, is limited [20, 21, 22, 23]. There are no specific heart failure treatment recommendations for osimertinib‐associated cardiac dysfunction, and therefore a general approach similar to trastuzumab‐related cardiac dysfunction is recommended in line with the 2021 ESC HF guidelines and the 2022 AHA/ACC/HFSA guidelines. For patients with reduced LVEF and NYHA class II to III symptoms, Class I guideline recommendations include initiation and uptitration of BB, RAS blockade, preferably with an angiotensin receptor‐neprilysin inhibitor (ARNI), mineralocorticoid receptor antagonists (MRA), and SGLT2 inhibitors to reduce morbidity and mortality [15]. Additionally, the patient should be initiated on loop diuretics due to evidence of volume overload.

Emerging data suggest that SGLT2i may be cardioprotective in patients undergoing cancer therapy with a reduced LVEF even in the absence of an established diagnosis of DM. Furthermore, these agents appear to have cardioprotective benefits against CTRCD [24]. Although limited data exist on the temporary discontinuation of osimertinib and subsequent rechallenge at a lower dose if HF improves, this strategy should be considered in clinical practice especially in cases where there is a good cancer response to targeted therapy [21, 25].

An important aspect of this case is the lack of baseline CV risk assessment, including Echo, in a patient with established CAD prior to initiation of cancer therapy. The optimal monitoring strategy during treatment with osimertinib has not been established but it is clear that a baseline assessment would be very useful and informative. The rate of major adverse cardiac events is much higher in patients with lung cancer than, for example, breast cancer, within 4 years of initiating treatment [26]. Additionally, many lung cancer treatments are known to have important potential CV toxicities [27]. During treatment, the patient developed symptomatic HF. However, it is not known if the reduced LVEF was related to osimertinib or was already present at the onset of cancer treatment. In fact, osimertinib has been associated with LV systolic dysfunction, perhaps related to the close association of EGFR receptor to HER2 (EGFR2) that this drug targets [1]. Per the ESC CO Guidelines, obtaining echo is strongly recommended at least in part to determine if cardioprotective therapy might be beneficial to prevent the development of CTRCD and to allow the most effective cancer therapy to be delivered. In general, baseline CV risk assessment is recommended (IC) for patients starting on EGFR Inhibitors.

Osimertinib is recommended as a first‐line treatment for patients with metastatic non‐small‐cell lung cancer (NSCLC) harboring common EGFR mutations, such as exon 19 deletions. The NCCN guidelines also note that osimertinib with chemotherapy (cisplatin/carboplatin + pemetrexed) is an alternative option for some patients, particularly those with high‐risk features (e.g., p53 mutations or presence of brain metastases). In the adjuvant setting, osimertinib is recommended for patients with resected stage IB‐IIIA EGFR mutation‐positive NSCLC to reduce the risk of disease recurrence. However, there is limited guidance in the NCCN or ASCO guidelines on cardiac optimization and testing pre‐treatment or monitoring for cardiotoxicity during treatment (Table 3) [28]. Osimertinib has been associated with the potential for QT prolongation, arrhythmias, and HF when compared with other targeted treatments for NSCLC or other agents known to prolong QT intervals [29].

**TABLE 3:** Recommended baseline assessments for Osimertinib.

	ASCO	NCCN	ESC/IC‐OS Cardio‐oncology guidelines
Baseline assessment	None	None	Baseline ECG (Class I, LOE C) Echo (Class I, LOE B)
Baseline cardiac biomarkers	None	None	None
Cardiac monitoring during therapy	None	None	Every 3 months (Class IIa, LOE B)
Recommended cardioprotection	None	None	None
Continuation of osimertinib with symptom control	None	None	Permissive cardiotoxicity

*Note:* BNP, brain natriuretic peptide Class I (Evidence and/or general agreement that a given treatment or procedure is beneficial, useful, effective), Class II (Conflicting evidence and/or a divergence of opinion about the usefulness/efficacy of the given treatment or procedure, IIa: weight of evidence/opinion is in favor of usefulness/efficacy), Class III: Evidence or general agreement that the given treatment or procedure is not useful/effective and in some cases maybe harmful. LOE A: Data derived from multiple randomized clinical trials or meta‐analyses. LOE B (Data derived from a single randomized clinical trial or large nonrandomized studies). LOE C (Consensus of opinion of the experts and/or small studies, retrospective studies, registries).

Abbreviations: HF, heart failure; LVEF, left‐ventricular ejection fraction.

### 3.3 Sarcoma and Breast Cancer

A 38‐year‐old female with a documented Li‐Fraumeni syndrome was recently diagnosed with cT2 (2.5 cm) N0M0, grade 3, ER/PR/HER2 negative (triple negative) infiltrating ductal carcinoma. She was previously treated for right femur osteosarcoma, diagnosed at age 15, and treated with doxorubicin (300 mg/m^2^ total dose) and ifosfamide for a total of 6 cycles, followed by surgical resection and radiation therapy to her right leg. She has been in remission since then but was lost to follow up from the survivorship program 5 years ago.

As part of the evaluation for systemic therapy, an echo revealed an LVEF of 51% with a mildly dilated left ventricle (LVEDVi of 67 mL/m^2^, normal < 61 mL/m^2^) and mildly reduced global longitudinal strain of −15% (normal GLS is between −17% and −22%, and this is abnormal since GLS represents myocardial shortening). The patient reports being able to perform daily activities but does get short of breath going upstairs or an incline. On examination, the BP was 155/87 mmHg and HR 92 bpm, and she had no signs of decompensated HF. Her oncology team would like to offer her neoadjuvant therapy with KEYNOTE‐522 regimen consisting of carboplatin and taxol weekly × 12 followed by doxorubicin/cyclophosphamide every 3 weeks × 4, all given concurrently with pembrolizumab every 3 weeks [30].

At this juncture, are there any special considerations or treatment that could reduce her risk of CTRCD and allow her cancer treatment to proceed without interruption?

- The administration of dexrazoxane prior to doxorubicin is recommended to mitigate cardiotoxicity, and maintaining blood pressure below 130/80 mmHg using cardioprotective antihypertensive agents is advisable.
- Cardiac biomarkers such as NT‐proBNP or troponin are not effective in further refining CV risk assessment.
- ECG monitoring at intervals of 4–6 weeks is adequate for the detection of CV complications.
- Concerns regarding CTRCD are diminished due to the younger age of the patient.

The correct answer is A. This patient has presumed mild symptomatic CTRCD at baseline prior to planned chemotherapy since she is mildly symptomatic (Table 4) and has an abnormal GLS. According to the CTCAE Version 5 criteria, the patient does not meet grade 2 criteria for LVEF reduction as she did not have a 10%–19% drop in LVEF from baseline and her LVEF is 51%. The other category in CTCAE of LVSD does not apply at this juncture and this patient would be considered symptomatic from HF but grade 2 at baseline (Table S2). In addition, she is very high risk for worsening CTRCD during anthracycline‐based treatment because of prior anthracycline therapy “300 mg/m^2^ total dose.” With the planned additional anthracycline therapy, she will surpass the commonly accepted lifetime cumulative dose threshold of 450–550 mg/m^2^.

**TABLE 4:** Recommended diagnostic tests for patients suspected of having heart failure [31].

Recommendations	Class	Level
BNP/NT‐proBNP	I	B
12‐lead ECG	I	C
Transthoracic echocardiography	I	C
Standard blood tests for comorbid conditions; HbA1c, lipid profile, and iron levels (transferrin saturation and ferritin).	I	C

One of the potential strategies to ameliorate the elevated risk of CTRCD is utilizing dexrazoxane since planned chemotherapy includes additional anthracycline exposure [32]. Dexrazoxane has been shown to inhibit DNA topoisomerase IIB–anthracycline mediated double stranded DNA damage and minimize the production of free radicals. Historically, most of the benefits shown were demonstrated in patients with metastatic breast cancer subjected to a high cumulative dose of anthracyclines. A previous trial of breast cancer patients who have received dexrazoxane experienced fewer cardiac events than those who did not receive dexrazoxane (13% vs. 39%). However, the definition of cardiac events utilized in the study included asymptomatic reductions in LVEF measured either by MUGA scan or echo and reported fewer events of developing HF (1% vs. 11%) [33]. A concern arose that dexrazoxane might attenuate a complete response to chemotherapy and enhance tumor progression, but over the years, this concern appeared to be unsubstantiated [34]. Furthermore, a recent prospective trial in patients with sarcoma revealed that upfront dexrazoxane was not only cardioprotective but appeared to extend progression free survival in patients who were receiving high doses of anthracycline [35]. Further, a meta‐analysis on the use of dexrazoxane in breast cancer confirmed that there was a decreased risk of developing HF (RR: 0.19; *p* < 0.001) without affecting the oncological response (RR: 1.10; *p* = 0.62), overall survival (HR: 1.01; *p* = 0.92), and progression‐free survival (HR: 0.97; *p* = 0.81) [36].

Risk stratification of patients at baseline and during potentially cardiotoxic therapy as in the above clinical case is imperative. Utilizing cardiac biomarkers such as NT‐proBNP is an easy and effective method for risk stratification. A recent prospective study demonstrated that a BNP level greater than 100 pg/mL, measured before and within 24 h of anthracycline infusion, was highly predictive of subsequent cardiac events. Notably, approximately 10% of the study cohort experienced cardiac events, all of whom had BNP levels exceeding 100 pg/mL [37]. In a separate longitudinal study involving approximately 1200 adult survivors of childhood cancer, primarily lymphoma and bone cancers, who underwent anthracycline chemotherapy and/or chest radiation therapy (RT), nearly 25% of participants exhibited abnormal NT‐proBNP levels after therapy that surpassed age and sex‐specific thresholds. A dose‐dependent relationship was observed between the cumulative dose of anthracyclines, chest RT, and elevated NT‐proBNP levels (*p* < 0.0001). Over a median follow‐up of 5.4 years, survivors with abnormal NT‐proBNP levels, and no baseline history of cardiomyopathy, faced an increased risk of major adverse cardiac events (HR 1.75, 95% CI 1.04–2.94). This risk was particularly pronounced for the development of cardiomyopathy, with a twofold increase (HR 2.28, 95% CI 1.28–4.08), independent of baseline risk factors such as age, sex, HTN, and obesity [38]. In several studies, troponins were found not to be of any significant value [38, 39].

HTN represents an often‐overlooked modifiable risk factor, particularly among cancer survivors. Adequate blood pressure control is important to reduce the incidence of subsequent CV complications. Data from the St. Jude Lifetime Cohort Study demonstrate that the prevalence of HTN in survivors of childhood cancers is substantial, exceeding 70% by the age of 50. Furthermore, among individuals with normotensive readings at baseline, 5% developed HTN, and 21% developed prehypertension over a mean follow‐up period of 3.6 years, underscoring the need for ongoing surveillance and early intervention [40]. Cancer survivors exposed to anthracyclines who also have HTN are at markedly elevated risk for developing HF [41, 42]. Finally, annual screening for modifiable CV risk factors is a Class I, Level C recommendation for asymptomatic adult survivors of childhood and adolescent cancer who were exposed to anthracycline therapy [1].

### 3.4 Lymphoma

A 45‐year‐old male survivor of Hodgkin Lymphoma presents with progressive shortness of breath over the past 2–3 months. His symptoms are most pronounced during exertion, such as brisk walking or climbing stairs at work. He was treated for lymphoma at age 21 with 4 cycles of ABVD therapy followed by mantle radiation (40 Gy fractions for bulky mediastinal disease). He denies any symptoms at rest and his physical examination reveals no signs of HF. He has no other CV risk factors. An echo reveals LVEF of 45% with suggestion of wall motion abnormalities in the anterior wall. An exercise nuclear stress test reveals a reversible ischemic defect in the anteroseptal and apical segments consistent with CAD in the left anterior descending (LAD) artery distribution. He was scheduled for coronary angiogram with baseline complete blood count showing a hemoglobin 11.2 (g/dL), platelet count of 70 (10*^3^/μL) and white blood cell count of 5.4 (10*^3^/μL) with abnormal differential revealing decreased neutrophils, increased monocytes, and the presence of dysplastic cells. He underwent percutaneous coronary intervention to the proximal segment of the LAD and was placed on dual antiplatelet therapy with aspirin and clopidogrel. He was seen in 1 month in the cardiology clinic, and platelet count has decreased to 55 (10*^3^/μL) while on dual antiplatelet therapy. He did not experience any bleeding symptoms such as hemoptysis or melena.

What should be the next steps in managing this patient?

- He should continue dual antiplatelet therapy for 6 months at least.
- Refer the patient to a hematologist and consider bone marrow evaluation for myelodysplasia or secondary leukemia.
- Repeat CBC in 4–6 weeks and consider stopping aspirin while continuing clopidogrel and awaiting a hematological consultation.
- Perform thromboelastogram and if normal clotting continue dual antiplatelet therapy.

The correct answer is C. The patient presented with stable angina, where shortness of breath serves as an angina equivalent. He underwent an ischemic evaluation showing significant LAD lesion warranting a PCI given the decline in LVEF. This patient had mild symptomatic CTRCD by the ESC/IC‐OS criteria, based on his reduced LVEF and previous doxorubicin treatment, and his CAD was predominantly a result of mediastinal radiation received years earlier. Mantle radiation is a well‐known risk factor for premature and accelerated atherosclerosis, especially ostial lesions of the left or right coronary artery, particularly if the mean heart dose exceeds 25 Gy [43, 44].

The patient's complete blood count findings are concerning for therapy‐related myelodysplastic syndrome or acute myeloid leukemia in the context of his previous ABVD chemotherapy. Therapy‐related MDS accounts for approximately 10%–20% of all MDS cases and is typically associated with higher risk cytogenetic features, a poorer prognosis, and reduced overall survival [45, 46] In this context, optimizing CV status is essential to improve long‐term outcomes. However, the progressive decline in platelet count to 55 × 10^3^/μL 1 month after coronary intervention poses a clinical dilemma, particularly regarding the safety and continuation of dual antiplatelet therapy. According to SCAI Expert Consensus Statement, prasugrel, ticagrelor, and GP (Glycoprotein) IIb/IIIa inhibitors (such as tirofiban and eptifibatide) should be avoided in patients with platelet counts below 50,000/μL. Clopidogrel‐based dual antiplatelet therapy may be considered when platelet counts are between 30,000 and 50,000/μL, while aspirin may be used when counts exceed 10,000/μL [47] Furthermore, findings from the prospective PROTECT‐OCT registry suggest that intravascular imaging with optical coherence tomography may aid in identifying patients at low risk for stent thrombosis, potentially supporting early discontinuation of dual antiplatelet therapy [48].

An equally important consideration in this case is the need for long‐term CV surveillance following exposure to chemotherapy and radiation therapy. The 2022 ESC Cardio‐Oncology guidelines recommend performing a baseline CV risk assessment, including estimation of 10‐year risk for fatal and nonfatal CV disease using an appropriately validated 10 year CV risk score, for example, SCORE2 (Systematic Coronary Risk Evaluation 2) or SCORE2‐OP (Systematic Coronary Risk Evaluation 2‐Older Persons) (Class I, Level of Evidence B). In patients with a history of CVD undergoing radiation involving the heart, baseline echocardiography is also advised (Class IIa, Level of Evidence C) [1]. The ICOS expert consensus further emphasizes a comprehensive approach that includes detailed history and physical examination, review of prior CT imaging for atherosclerotic calcifications, optimization of CV risk factors, and acquisition of baseline ECG and echo. Follow‐up with an echo and ischemic evaluation is also recommended at 5 years posttreatment [43]. Surveillance scans to confirm ongoing remission may also reveal coronary artery calcifications, which hold prognostic value as predictors of cardiovascular morbidity and mortality [49, 50].

### 3.5 Melanoma

A 74‐year‐old male with a history of HTN, HL, and stage IIIb melanoma (BRAF V600 positive), incompletely resected 4 months ago, presents with progressive symptoms during adjuvant immunotherapy. He has received two cycles of nivolumab (1 mg/kg) and ipilimumab (3 mg/kg) over the past 2 months, with the most recent treatment administered 4 weeks ago. He reports a 1‐week history of worsening dyspnea, fatigue, palpitations, and generalized muscle weakness. Over the past 2–3 days, he has experienced exertional dyspnea with minimal activity and dysphagia, primarily for solids. Additionally, he reports proximal muscle weakness affecting neck flexion and ambulation, accompanied by blurry vision for 1 week.

The patient's vital signs include a heart rate of 43 bpm, blood pressure of 97/50 mmHg, respiratory rate of 24/min, and oxygen saturation of 93% on 2 L/min of nasal cannula. Physical examination reveals ptosis and bilateral proximal muscle weakness in the upper arms and thighs. Cardiac examination is unremarkable, with no murmurs, rubs, or gallops. Lung auscultation reveals clear fields but diminished breath sounds at the posterior bases. There is no jugular venous distension or lower extremity edema.

Laboratory results reveal a markedly elevated creatine kinase level of 3562 U/L (normal < 140 U/L) and significantly elevated liver enzymes, including aspartate aminotransferase at 235 U/L (normal < 33 U/L) and alanine transaminase at 345 U/L (normal < 36 U/L). High‐sensitivity troponin‐T is critically elevated at 5452 ng/L (normal < 20 ng/L), with persistently elevated levels over the following 24 h.

A 12‐lead electrocardiogram shows complete heart block and a ventricular escape rhythm at 44 bpm, with a QRS duration of 140 msec. Transthoracic echocardiography reveals a normal LVEF of 62% with no wall motion abnormalities or pericardial effusion. Invasive coronary angiography demonstrates no obstructive coronary artery disease. Cardiac magnetic resonance (CMR) imaging has been ordered and is pending.

What is the most important clinical diagnosis and treatment that should be considered at this point?

- This is likely an overlap of ICI myositis and myocarditis; it is potentially fulminant, so we should immediately administer high dose steroids.
- CMR is highly sensitive in evaluating ICI myocarditis; if negative, myocarditis is unlikely and endomyocardial biopsy is not indicated.
- ICI associated myocarditis is unlikely given the patient has normal LVEF and no clear evidence of HF on exam.
- The onset of ICI associated myocarditis occurs typically more than 6 months after the first administration of ICI.

The correct answer is A. The patient is presenting with signs concerning for fulminant ICI myocarditis, given the recent initiation of dual ICI treatment, presence of cardiac symptoms, overlapping symptoms of myositis, and myasthenia gravis like syndrome, with elevations in cardiac and skeletal muscle biomarkers [51]. According to ASCO guidelines, for patients with grade ≥ 2 toxicity, early initiation of high‐dose corticosteroids (1–2 mg/kg/day of prednisone, administered orally or intravenously) is recommended, as it is likely to provide benefit without significant adverse effects. In cases presenting with features of myasthenia gravis (without clinical evidence of myocarditis), treatment can begin with oral prednisone at 0.5 mg/kg, with the addition of intravenous immunoglobulin or plasmapheresis considered for more severe cases [52]. Similarly, the NCCN guidelines recommend initiating high‐dose steroids, such as intravenous methylprednisolone at 1 g/day for 3–5 days, with a transition to oral prednisone (1 mg/kg/day) once the patient shows improvement and is clinically stable. If no significant improvement is observed within 24–48 h of steroid therapy, additional immunosuppressive treatments should be considered [53]. Finally, a temporary pacemaker should be placed until complete heart block resolves with treatment as it is often reversible [54].

According to the IC‐OS consensus statement, this patient meets the criteria for grade 4 ICI myocarditis, signifying severe disease [2, 55]. A recent study by Zadok et al. highlighted the significant cardiovascular risks associated with severe ICI myocarditis. Patients with severe myocarditis exhibited a markedly higher cumulative incidence of 1‐year CV mortality compared to those with nonsevere myocarditis (HR: 6.52; *p* < 0.001). This increased mortality was primarily driven by a heightened death rate within the first 1–2 months following diagnosis. However, the study also found that 1‐year all‐cause mortality did not significantly differ between patients with severe myocarditis and those with non‐severe or negative cases (*p* = 0.74), underscoring the importance of early recognition and management of severe cases to mitigate short‐term risks [56].

Rechallenging a patient with ICIs following ICI myocarditis remains a controversial topic, with guidelines providing limited clarity, particularly in severe cases. According to ASCO guidelines, the appropriateness of rechallenging is uncertain in both ICI myocarditis and other severe immune‐related adverse events. A potential strategy is to consider using a single‐agent ICI rather than dual therapy [52, 57]. In contrast, NCCN guidelines recommend permanent discontinuation of ICIs in cases of grade 2–4 myocarditis. When rechallenge is attempted, it should only be pursued after the patient has recovered to grade 1 severity or lower (Table 5). However, there are no specific recommendations on selecting patients for rechallenge, even in cases where the myocarditis workup is negative [56]. The risks associated with rechallenge should be discussed in details with the patients and weighted against the risk of recurrence. An alternative therapeutic option for this patient, given the presence of a BRAF V600‐positive mutation, is the combination of dabrafenib and trametinib [58]. However, this approach presents challenges, particularly in the context of recent immune‐related cardiomyopathy, as the dabrafenib/trametinib combination itself is associated with a well‐documented risk of cardiomyopathy [59, 60]. This patient would be considered high risk and requires close monitoring after starting dabrafenib/trametinib, for example, an echo and measurement of cardiac biomarkers at baseline and at 6–8 weeks after starting dabrafenib/trametinib and 3 monthly thereafter. This potential overlap of cardiac toxicities necessitates careful patient selection, baseline cardiovascular evaluation, and multidisciplinary discussion with close monitoring during therapy.

**TABLE 5:** Societal guidelines on the management of ICI–associated myocarditis and recommendations for rechallenging patients with ICIs.

Guidelines entity	Grading	Treatment	Rechallenge
ASCO	4	1—Initiation of high‐dose corticosteroids (1–2 mg/kg/d of prednisone, oral or IV depending on symptoms). 2—For new conduction delay, consider a pacemaker. 3—Consider the addition of either mycophenolate, infliximab, or antithymocyte globulin in patients refractory to steroids. 4—Consider abatacept or alemtuzumab as additional immunosuppression in life‐threatening cases.	1—Uncertain 2—Consider single‐agent ICI rather than dual therapy
NCCN	4	1—High‐dose steroids (methylprednisolone) 1 g/day IV for 3–5 days. 2—If responding, switch to oral prednisone (1 mg/kg/day), then taper slowly over 6–12 weeks. 3—If refractory, initiate additional immunosuppression: Abatacept, Alemtuzumab. Antithymocyte globulin, Infliximab, IVIG, methotrexate or Mycophenolate	Permanent discontinuation in cases of grades 2–4 myocarditis
ESC/IC‐OS	Fulminant	1—If the suspicion is high, administer steroids while undergoing workup. 2—Methylprednisolone 500–1000 mg intravenous bolus once daily (minimum 3 days) then transition to oral prednisolone (1 mg/kg/day) if patient is recovering. 3—In steroid refractory cases, consider second‐line immunosuppressive agents	Recommend a multidisciplinary team discussion on a case by case

## 4 Conclusion

CTRCD is a well‐established complication of oncological treatments that demands an integrated, proactive multidisciplinary approach involving cardiology, hematology, and oncology teams. Despite the availability of comprehensive cardio‐oncology guidelines, their translation into daily clinical practice remains challenging. This consensus statement provides pragmatic, case‐based applications of evidence‐based definitions and treatment recommendations, tailored to a spectrum of malignancies and associated cardiovascular presentations. By standardizing CTRCD risk assessment, surveillance, and management, we aim to preserve oncologic treatment intensity, minimize cardiovascular morbidity, and improve overall outcomes. Ultimately, fostering interdisciplinary collaboration and equipping noncardiology providers with user‐friendly tools is essential to minimize cardiovascular complications as a barrier to optimal cancer care.

## Author Contributions

**Abdelrahman Ali:** conceptualization (equal), data curation (equal), methodology (equal), project administration (equal), resources (equal), writing – original draft (lead), writing – review and editing (lead). **Suparna C. Clasen:** conceptualization (supporting), methodology (supporting), writing – original draft (supporting), writing – review and editing (supporting). **Anne Blaes:** conceptualization (supporting), writing – review and editing (supporting). **Stephen Casselli:** writing – original draft (supporting), writing – review and editing (supporting). **Anita Deswal:** conceptualization (supporting), writing – original draft (supporting), writing – review and editing (supporting). **Susan Halli Demeter:** writing – original draft (supporting), writing – review and editing (supporting). **Gregory Durm:** writing – original draft (supporting), writing – review and editing (supporting). **Anecita Fadol:** writing – original draft (supporting), writing – review and editing (supporting). **Alessandra Ferrajoli:** writing – original draft (supporting), writing – review and editing (supporting). **Michael G. Fradley:** writing – review and editing (supporting). **Joerg Herrmann:** conceptualization (equal), methodology (equal), writing – original draft (supporting), writing – review and editing (supporting). **Borja Ibanez:** writing – original draft (supporting), writing – review and editing (supporting). **Sue Koob:** writing – original draft (supporting), writing – review and editing (supporting). **Bogda Koczwara:** writing – original draft (supporting), writing – review and editing (supporting). **Kasey J. Leger:** conceptualization (equal), writing – original draft (supporting), writing – review and editing (supporting). **Jennifer E. Liu:** conceptualization (equal), writing – original draft (supporting), writing – review and editing (supporting). **Teresa López‐Fernández:** conceptualization (equal), methodology (equal), writing – original draft (supporting), writing – review and editing (supporting). **Alexander R. Lyon:** conceptualization (equal), methodology (equal), writing – original draft (supporting), writing – review and editing (supporting). **N. G. Choon Ta:** conceptualization (equal), writing – original draft (supporting), writing – review and editing (supporting). **John Teerlink:** writing – original draft (supporting), writing – review and editing (supporting). **Eric H. Yang:** conceptualization (equal), methodology (equal), writing – original draft (supporting), writing – review and editing (supporting). **Susan Dent:** conceptualization (supporting), methodology (supporting), resources (supporting), writing – original draft (supporting), writing – review and editing (supporting). **Daniel Lenihan:** conceptualization (equal), methodology (equal), project administration (equal), resources (equal), visualization (equal), writing – original draft (equal), writing – review and editing (equal).

## Funding

The authors have nothing to report.

## Conflicts of Interest

Dr. Deswal serves as a consultant for Bayer. Dr. Leger is a consultant for Servier and Jazz Pharmaceuticals. All other authors declare no conflicts of interest.

## Supporting information


**Data S1:** cam471682‐sup‐0001‐DataS1.docx.

## Data Availability

All data which led to the formulation of the recommendations presented is available publicly and has been published in online resources.
