# Supplementary material for: Standardizing the Clinical Approach to Cancer Therapy‐Related Cardiac Dysfunction: Applying Cardio‐Oncology Guidelines as a Practical Tool for Hematology and Oncology Providers
Source: Cancer Med. 2026 Mar 27;15(4):e71682. doi: 10.1002/cam4.71682 (PMC13140896; doi:10.1002/cam4.71682)
Supplement: Supplementary file 1 — Data S1: cam471682‐sup‐0001‐DataS1.docx. [file CAM4-15-e71682-s001.docx]

**Supplementary Table 1. Screening Criteria for important CV adverse events.**

| 1. **Symptomatic Heart Failure Defined as:**   (Demonstrated by at least one item in two of the three following categories) | | |
| --- | --- | --- |
| **Symptoms of HF** | | |
| Yes | No | Paroxysmal Nocturnal Dyspnea |
| Yes | No | Shortness of Breath |
| Yes | No | Swelling |
| Yes | No | Fatigue |
| Yes | No | Orthopnea |
| Yes | No | Weight Gain |
| **Physical Findings** | | |
| Yes | No | Jugular Venous Distention |
| Yes | No | Crackles |
| Yes | No | Edema |
| Yes | No | S3 |
| **Diagnostic Tests** | | |
| Yes | No | Pulmonary Edema on CXR |
| Yes | No | BNP >100pg/ml, *if Yes: BNP*: ____________ NT-ProBNP > 300ng/ml, If Yes: NT-ProBNP: ---------- |
| 2. Acute coronary syndrome is defined as 2 of 3 clinical findings of chest pain, troponin I elevation and/or ECG changes. | | |
| 3. Sudden cardiac death defined as death within 24 hours of proteasome inhibitor treatment without obvious cancer or disease progression. | | |
| 4. Symptomatic arrhythmia (bradycardia, AV block, VT, atrial fibrillation) requiring treatment. | | |
| 5. Arterial and/or deep venous thromboembolism. | | |
| 6. Pulmonary HTN defined as tricuspid regurgitation >3 m/s. | | |
| 7. CTCAE v4.0- Grade III HTN defined as systolic BP > 160 mm Hg or diastolic BP > 100 mm Hg; medical intervention indicated; more than one drug or more intensive therapy than previously used indicated. | | |
| 8. CTCAE v4.0- Grade IV HTN defined as life-threatening consequences (e.g., malignant hypertension, transient or permanent neurologic deficit, hypertensive crisis); urgent intervention indicated. | | |

HF: heart failure, CXR: chest x-ray, BNP: brain natriuretic peptide, VT: ventricular tachycardia, HTN: hypertension, CTCAE: common terminology criteria for adverse events

**Supplementary Table 2 - Definitions for Cancer Therapy Related Cardiac Dysfunction**

|  | **Definition** |
| --- | --- |
| Definition of Chemotherapy-induced Cardiotoxicity ^(2)^ | Any of the following:  (1) reduction of LVEF, either global or more severe in the interventricular septum.  (2) symptoms of congestive heart failure  (3) signs associated with heart failure (HF), such as S3 gallop, tachycardia, or both  (4) reduction in LVEF from baseline by >_5% to <55% in the presence of signs or symptoms of HF, or a reduction in LVEF by >_10% to <55% without signs or symptoms of HF |
| NYHA Classification | Class I: No symptoms. Class II: Mild symptoms and slight limitation during ordinary activity. Class III: Marked limitation due to symptoms, even with less than ordinary activity. Class IV: Symptoms at rest. |
| ACCF/AHA Stages of HF | Stage A: At high risk for HF but without structural disease or symptoms of HF. Stage B: Structural heart disease but without signs or symptoms of HF. Stage C: Structural heart disease with prior or current symptoms of HF. Stage D: Refractory HF requiring specialized interventions. |
| CTCAE Version 5 - Ejection Fraction Decreased | Grade 2: Resting LVEF 50-40%; 10-19% drop from baseline. Grade 3: Resting LVEF 39-20%; ≥20% drop from baseline. Grade 4: Resting LVEF < 20%. |
| CTCAE Version 5 - LV Systolic Dysfunction | Grade 3: Symptomatic due to drop in LVEF responsive to intervention. Grade 4: Refractory or poorly controlled HF due to drop in LVEF; intervention such as ventricular assist device, intravenous vasopressor support, or heart transplant indicated.  Grade 5: Death |
| CTCAE Version 5 - Heart Failure | Grade 1: Asymptomatic with laboratory (e.g., BNP) or cardiac imaging abnormalities. Grade 2: Symptoms with moderate activity or exertion. Grade 3: Symptoms at rest or with minimal activity or exertion; hospitalization; new onset of symptoms. Grade 4: Life-threatening consequences; urgent intervention indicated (e.g., continuous IV therapy or mechanical hemodynamic support). |

**Contribution**Abdelrahman Ali: Conceptualization (Equal); Data curation (Equal); Methodology (Equal); Project administration (Equal); Resources (Equal); Writing – original draft (Lead); Writing – review & editing (Lead).

Suparna Clasen: Conceptualization (Supporting); Methodology (Supporting); Writing – original draft (Supporting); Writing – review & editing (Supporting).

Anne Blaes: Conceptualization (Supporting); Writing – review & editing (Supporting).

Stephen Casselli: Writing – original draft (Supporting); Writing – review & editing (Supporting).

Anita Deswal: Conceptualization (Supporting); Writing – original draft (Supporting); Writing – review & editing (Supporting).

Susan Halli Demeter: Writing – original draft (Supporting); Writing – review & editing (Supporting).

Greg Durm: Writing – original draft (Supporting); Writing – review & editing (Supporting).

Anecita Fadol: Writing – original draft (Supporting); Writing – review & editing (Supporting).

Allessandra Ferrajoli: Writing – original draft (Supporting); Writing – review & editing (Supporting).

Michael G. Fradley: Writing – review & editing (Supporting).

Joerg Herrmann: Conceptualization (Equal); Methodology (Equal); Writing – original draft (Supporting); Writing – review & editing (Supporting).

Borja Ibanez: Writing – original draft (Supporting); Writing – review & editing (Supporting).

Sue Koob: Writing – original draft (Supporting); Writing – review & editing (Supporting).

Bogda Koczwara: Writing – original draft (Supporting); Writing – review & editing (Supporting).

Kasey Leger: Conceptualization (Equal); Writing – original draft (Supporting); Writing – review & editing (Supporting).

Jennifer E. Liu: Conceptualization (Equal); Writing – original draft (Supporting); Writing – review & editing (Supporting).

Teresa López-Fernández: Conceptualization (Equal); Methodology (Equal); Writing – original draft (Supporting); Writing – review & editing (Supporting).

Alexander R. Lyon: Conceptualization (Equal); Methodology (Equal); Writing – original draft (Supporting); Writing – review & editing (Supporting).

Choon Ta Ng: Conceptualization (Equal); Writing – original draft (Supporting); Writing – review & editing (Supporting).

John Teerlink: Writing – original draft (Supporting); Writing – review & editing (Supporting).

Eric Yang: Conceptualization (Equal); Methodology (Equal); Writing – original draft (Supporting); Writing – review & editing (Supporting).

Susan Dent: Conceptualization (Supporting); Methodology (Supporting); Resources (Supporting); Writing – original draft (Supporting); Writing – review & editing (Supporting).

Daniel Lenihan: Corresponding author; Conceptualization (Equal); Methodology (Equal); Project administration (Equal); Resources (Equal); Visualization (Equal); Writing – original draft (Equal); Writing – review & editing (Equal).
